# Cross sectional dopamine transporter imaging and myocardial sympathetic scintigraphy study in patients with dementia with Lewy bodies

**DOI:** 10.1002/alz.087839

**Published:** 2025-01-09

**Authors:** Shigeki Hirano, Zhihui Tang, Yume Koizumi, Michiko Izumi, Yoshihisa Kitayama, Kosuke Yamagishi, Mitsuyoshi Tamura, Ai Ishikawa, Kouichi Kashiwado, Takashi Iimori, Hiroki Mukai, Hajime Yokota, Takuro Horikoshi, Takashi Uno, Satoshi Kuwabara

**Affiliations:** ^1^ Department of Neurology, Graduate School of Medicine, Chiba University, Chiba Japan; ^2^ Department of Radiology, Chiba University Hospital, Chiba Japan; ^3^ Department of Diagnostic Radiology and Radiation Oncology, Graduate School of Medicine, Chiba University, Chiba Japan

## Abstract

**Background:**

Dopamine transporter (^123^I‐FP‐CIT) single‐photon emission tomography (SPECT) and ^123^I‐meta‐iodobenzylguanidine (^123^I‐MIBG) image play roles as indicative biomarkers in diagnosing patients with dementia with Lewy bodies (DLB). Brain‐ and body‐first subtypes of DLB were hypothesized implying that subset of DLB may have normal ^123^I‐FP‐CIT or ^123^I‐MIBG results, respectively. The purpose of this study was to explore the diagnostic sensitivity of two combination imaging modalities (^123^I‐FP‐CIT SPECT and ^123^I‐MIBG image) in patients with DLB and examine the clinical difference between brain‐ and body‐first subtype.

**Method:**

Possible DLB patients, defined by the fourth consensus DLB criteria, who underwent both ^123^I‐FP‐CIT SPECT and ^123^I‐MIBG image was retrospectively collected. Semi‐automated software and their results were utilized to define abnormality for both scans. Demographic data, cognition, motor, and core features were compared between the brain‐first and body first DLB subgroups.

**Result:**

Of the 114 patients with DLB, 66 underwent both scans. Mean (SD) age was 74.1 (7.1) years, male was predominant (62.1%), min‐mental state examination total score was 21.7 (4.8) and unified Parkinson’s disease rating scale motort subscore was 14.7 (4.8). Thirty‐six (54.5%) patients showed both abnormal scans and four patients (6.1%) were both normal scans. Seventeen patients (25.8%) showed abnormal ^123^I‐FP‐CIT result with normal ^123^I‐MIBG result (brain‐first DLB), nine patients (13.6%) showed normal ^123^I‐FP‐CIT result with abnormal ^123^I‐MIBG result (body‐first DLB). No clinical differences were observed between brain‐first and body‐first DLB subtypes.

**Conclusion:**

Approximately 40% of DLB patients displayed normal result in either image. Normal results of a single imaging test may not refute the possibility of DLB.

No clinical difference was observed between brain‐ and body‐first DLB subtypes.

